# Genomics and Ethics: The Case of Cloned and/or Transgenic Animals

**DOI:** 10.1002/cfg.243

**Published:** 2003-02

**Authors:** Béatrice de Montera

**Affiliations:** 1 Laboratoire de Biologie du Développement et Biotechnologies INRA Domaine de Vilvert 78 352 Jouy-en-Josas cedex France

## Abstract

The point of the present study is to illustrate and, if possible, promote the existing link between genomics and ethics, taking the example of cloned and transgenic animals. These ‘new animals’ raise theoretical and practical problems that concern applied ethics. We will explore more particularly an original strategy showing that it is possible, starting from philosophical questioning about the nature of identity, to use a genomic approach, based on amplification fragment length polymorphism (AFLP) and methylation-sensitive amplification polymorphism (MSAP) detection, to provide useful tools to define more rigorously what cloned animals are, by testing their genetic and epigenetic identity. We expect from the future results of this combined approach to stimulate the creativity of the philosophical and ethical reflection about the impact of biotechnology on animals, and to increase scientific involvement in such issues.


**Comparative and Functional Genomics**
*Comp Funct Genom* 2003; **4**: 26–30.
Published online in Wiley InterScience (www.interscience.wiley.com). **DOI:** 10.1002/cfg.243

**Conference Review**

### Genomics and ethics: the case of cloned and/or transgenic animals

Béatrice de Montera*
*Laboratoire de Biologie du Développement et Biotechnologies, INRA, Domaine de Vilvert, 78 352 Jouy-en-Josas cedex, France*

*Correspondence to:
Béatrice de Montera, Laboratoire de Biologie du Développement et Biotechnologies, INRA, Domaine de Vilvert, 78 352 Jouy-en-Josas cedex, France.
E-mail: bdemontera@tiscali.fr

Received: 7 September 2002
Accepted: 29 November 2002

### Abstract

**The point of the present study is to illustrate and, if possible, promote the existing link between genomics and ethics, taking the example of cloned and transgenic animals. These 'new animals' raise theoretical and practical problems that concern applied ethics. We will explore more particularly an original strategy showing that it is possible, starting from philosophical questioning about the nature of identity, to use a genomic approach, based on amplification fragment length polymorphism (AFLP) and methylation-sensitive amplification polymorphism (MSAP) detection, to provide useful tools to define more rigorously what cloned animals are, by testing their genetic and epigenetic identity. We expect from the future results of this combined approach to stimulate the creativity of the philosophical and ethical reflection about the impact of biotechnology on animals, and to increase scientific involvement in such issues. Copyright © 2003 John Wiley & Sons, Ltd.**

### Introduction

We aim to demonstrate the existing link between genomics and bioethics by a reflection of applied ethics that relies on the use of genomic tools.

Taking the case of cloned and/or transgenic animals, one can pose a philosophical question about the identity of such animals, identity being defined philosophically as a permanence or a resistance to external change. However, this definition is not sufficient to explain the particularity of these 'new beings' [9]. A genomic approach that would analyse the link between DNA structure and gene expression, by comparing genomes, could supply useful tools to clarify our understanding about cloned animals. It should be very interesting to build, on a scientific basis, some new philosophical questioning that could lead to the exploration of new pathways in applied ethics.

The cloning technique is difficult to classify because cloned animals are generally not considered as genetically modified. We can only note that cloning is a form of asexual reproduction that is unnatural in mammals. Transgenesis, by contrast, consists of a manipulation of the genome. Therefore, theoretical questions about the identity of cloned animals and transgenic ones are quite distinct, even if these techniques are not necessarily separate from a pragmatic point of view because using cloning via cell transformation remains the most profitable way of producing transgenic animals in cow and goat. The existence of cloned mammals obtained by nuclear transfer (which did not exist 7 years ago), in our case bovine clones, poses the problem of genetic identity in a different way to that of inbred animals. The initial purpose of cloning was to create genetically identical animals as so-called 'good experimental models', but the question is, are they truly genetically identical?

In addition, given that cloned animals have a different methylation pattern, one can suggest that it might be simplistic to assume that genetic identity is the only form of identity to examine. To apprehend the issues around identity in an original but scientific way, it is possible to use genomic tools such as AFLP [17] to test both genetic identity and methylation differences. Having scientific tools to study the identity of cloned animals should allow for improvement in the definition of these 'new animals', and therefore stimulate a more concrete

Copyright © 2003 John Wiley & Sons, Ltd.

reflection integrating their scientific, social and ethical status.

On the other hand, transgenesis poses the question of the physical resistance between species. This limit to manipulation could point to the existence of a species-specific identity.

These reflections lead us to two pragmatic questions: what is the utility of creating such new individualities, and what is the responsibility of the scientific community in such matters?

### Materials and methods

How can we go about answering the question of the identity of clones in a scientific way? If we consider that clones have no pathology, can we assert that they are normal and genetically identical, as they are expected to be? If we assume that sequencing the entire genome of each bovine clone is not feasible, we can handle the question of identity by using the AFLP technique [1] to study the genetic variability of normal clones. In order to find some answers to the question of the identity of these 'new animals', we are comparing the genomes of bovine clones derived from the same batch of cell cultures at the French National Institute of Agronomic Research (INRA). AFLP has not been tried on twinned animals, therefore we do not yet have an estimate of their degree of identity. Twins appear naturally (post-fertilization) by a process of spontaneous embryonic scission; clones, by contrast, are created by an artificial process before development, which avoids fertilization [13]. Would genomes originating from the same somatic genotype be identical in terms of their linear DNA structure, as are twins? The answer is normally 'yes' for twins (except in cases where spontaneous mutations occur), but needs to be tested particularly regarding clones, since clones derive from different nuclei that are artificially transferred into different cytoplasms. Would clones and twins be identical in terms of the function of their genome? Probably not, because of the differential methylation of genes in a given local environment. This question is particularly relevant regarding cloned animals because of the existence of variations in DNA (loci) methylation [11]. The questions of structural and functional identity of clones have therefore to be addressed in order to provide an estimate of the degree of difference.

DNA from cells obtained from ear skin and cultivated during one passage is purified with a DNA extraction kit (Qiagen, San Diego, USA). DNA is then digested with a rare site enzyme (*Eco*RI) and a frequent one (*Taq*I) to generate thousands of fragments of an average size of 256 bp. Each end of the amplified fragments is ligated with double-strand linkers to allow the amplification of all fragments with the same pair of primers. Two rounds of PCR are performed with primers containing one to three additional bases at each end, in order to reduce the number of possible amplifiable fragments. This allows the selective amplification of only a part of the fragment set corresponding to the specific sequence of each combination of primers. To permit the detection of amplified fragments on an automatic sequencer, the rare cutter primer side is fluorescently labelled. Of the potential $15 \times 10^9$ fragments generated by digestion of the bovine genome, only 50–150 will be detectable using each primer combination. The 83 possible combinations created by addition of 1–3 base pairs at the end of the amplification primers will provide access to 1%, at best, of the sequence information of the genome in order to check for the presence of point mutations or rearrangements. The AFLP technique is particularly well adapted to the detection of variations within a species. One usually uses the pair of primers that gives the highest number of differences, but our study requires the use of all possible primer combinations to obtain the best information about clone identity, even if it concerns only 1% of the genome.

If some polymorphisms are detectable, this study of the frequency of polymorphism in bovine clones will lead to the following question: what would the presence of a mutation between two clones of the same genetic background — same donor cell — signify? The possible presence of detectable mutations [10] in cloned animals could signify that these animals are not identical, as they were supposed to be, and this, in turn, could change our way of viewing the cloning technique. The response to the question of knowing whether cloning induces mutations depends on the results we will obtain.

The use of a variant of the AFLP technique, using a restriction enzyme sensitive to methylation called MSAP [18,19], would be interesting to test the epigenetic identity of clones by searching for aberrant methylation patterns that, in abnormal

Copyright © 2003 John Wiley & Sons, Ltd.

animals, could be linked to problematic cloning and associated pathologies [7].

### Discussion

### What are the existing ethical problems and questioning about cloned and/or transgenic animals, and what can we expect to learn from this scientific approach?

We must realize that clones and transgenic animals are not technological hypotheses, but concrete realities that pose very peculiar problems because they do not benefit from the protection of any proper legislation or ethics [2]. Until now, they have not been taken into consideration by a research ethics that has preferred action, instead of control, of the biotechnology used. This proposed scientific approach could help us to more precisely define and build an ethical status for these 'existing animals' which should never be considered only as 'hypothetical' ones [15]. The pragmatic question of what to do with cloned or transgenic animals should be linked to the question of the legitimacy of their conception and the issues around their living conditions.

### What are the concrete problems that have arisen with existing cloned animals?

First, we must deal with a technical weakness. Until now, bovine cloning, consisting of the fusion between a somatic cell and an enucleated oocyte, has had a very low success ratio of 1.5% (number of calves born : reconstituted embryos [5]). The problem is that it is difficult to find an explanation for such a bad ratio. Hence, there remains a 'grey area' in the cloning technique that should be clarified, perhaps via scientific studies like ours, which could serve as feedback on the technique.

This fuzzy technical context has heavy consequences during the gestation period. As we do not know how the nucleus is re-programmed, we cannot foresee the physiological consequences during embryo development [6]. Large offspring syndrome (LOS) in calves is the main pragmatic issue that scientists have to deal with and that poses ethical problems [20]. The foetus is indeed bigger than it should be and is often characterized by an asynchronous development of organs, e.g. enormous heart and kidneys, and small lungs [4]. This syndrome, for which scientists were not prepared because they have not solved all the theoretical problems around cloning, has resulted sometimes in catastrophes such as the breakdown of the uterus of some surrogate mothers. In this difficult context, the reaction of veterinarians and of animals caretakers is empirical.

Until now, decisions have been made using a cost vs. scientific interest balance. Creating cloned calves is expensive — 1.8 times more than for a calf produced using IVF [5] — and of great scientific interest, so the main purpose is to keep the foetus alive. This criterion is explicit, but there is another criterion, which is only implicit, which is the cost vs. animal suffering balance. In this case, cost symbolizes the global financial and scientific value of the calf, and it is compared to the cost of the surrogate mother's suffering. The description of what is usually done underlines the necessity of making decisions within an ethical framework. In this case, a conflict must be solved between two aspects of animal ethics, which are pathocentrism (where the value is the pain of the surrogate mother) and biocentrism (where the value is the life of the calf). On the other hand, we have to think about the possibility of creating a new status for bovine clones, because of their very particular care and sanitary conditions, which are much better than for other breeding animals.

Using genomic tools can perhaps lead, by clarifying the identity of clones, to an explanation for the weakness of the cloning technique and to an avoidance of the appearance of abnormalities during the development. Most importantly, it could allow a rigorous reflection about the theoretical and, more specifically, philosophical issues about the nature of animals created through cloning.

### What about the concrete problems that have arisen due to the existence of transgenic animals?

As we have already said, transgenesis poses a different theoretical problem from cloning itself. One of the ideas would be to create producers of proteins of therapeutic interest, e.g. in milk regarding bovine species. However, it seems largely to be premature to talk about therapeutic applications, considering the technical problems that currently occur (with the output ratio being extremely low).

Copyright © 2003 John Wiley & Sons, Ltd. 

The case of transgenic animals reveals the difficulty for a genome to tolerate the transfer of a foreign gene, and we know there can sometimes be severe consequences of the random insertion of a transgene [16]. Transgenic animals cannot be considered as mosaic organisms because they are not normally made up of cells with different genotypes derived from the same zygote [14], since they are considered as transgenic only if their cells are transgenic. A chimera is an organism that consists of cells derived from more than one individual; under this broad definition, transgenic animals could in some ways be considered as genetic chimeras, because their genome is formed from more than one genome. However, they are not cellular chimeras, because chimera cells are usually considered as being of different genotype and therefore chimeras are rather a subset of mosaics [14]. What could the difference be, then, between transgenic animals and animals that have undergone horizontal gene transfers during evolution, and could they be assimilated to chimeras? One answer could be that only selected genes, which are useful and do not cause a disturbance, have not been deleted from organisms through evolution after horizontal transfer. Through evolution, organisms have time to react to, and to adapt to, inherited internal changes, whereas the impact of a transgene, which is global for the organism, and brutal, does not allow for adaptation or further deletion. We could say, therefore, that transgenic animals are not protected by the evolutionary processes of gene loss and gain. The consequences of transgenesis on animals raise the question of specific identity, while cloning consequences raise the question of individual and genetic identity. However, the existence of transgenic animals is not only a theoretical subject, and we can consequently ask the practical question: what should be done with transgenic animals once they have been created? One can wonder if scientists always foresee the pragmatic consequences of their scientific projects when dealing with engineered animals, especially concerning mammals, and a species loved by the public, such as the bovine species.

In order to think about the conceptual issues of transgenesis and cloning, we can rely on philosophical concepts coming from Aristotle, such as form — or essence — and matter; concepts that, once reshaped, can help us to propose an explanation of the importance of the principles of identity and difference for an animal life. We could make the hypothesis of the transmission through generations of a form that transports a certain degree of identity and a certain degree of variation [12], corresponding in some way to the transmission of DNA and to the inheritance of epigenesis. Then the philosophical question would be: do genetic manipulation or cloning affect the essence — or identity — of an animal by introducing an abnormal degree of identity or difference? In what concerns us, we expect from our scientific study some new ingredients to sketch an answer to the question of identity concerning cloning.

We have the possibility now of summing up the ethical issues concerning this study. We find three questions with different natures:

1. The first question is an epistemological matter. Six years after Dolly's birth, there is a real necessity, from an ethical point of view, for scientific feedback on the cloning technique. It would be important to be able to give, thanks to genomic tools, the 'raison d'être' — the object (why) and goal (purpose) of what is done but also of what is created, since we must remember that we are talking about animals [3].
2. The second question is a question of animal ethics: whilst the theoretical problems of cloning and transgenesis on animals are still not solved, they infer practical problems which are the symbols of a technically and scientifically fuzzy context. The ethical question is, therefore: are cloned and transgenic animals supposed to benefit from a special ethical status, considering their outstanding symbolic and scientific status?
3. The third question is about research ethics concerning the use of mammals. Scientists find it difficult to define and justify what they are creating because of the absence of a clear definition of the status of these 'new beings' (which have a concrete existence) and because of their relationship with those animals [8]. We may think that the responsibility for thinking of the long-term utility is required for scientists during the construction of new projects. Helping to define more precisely the identity of clones would constitute a first step towards this fulfilling this responsibility.

As a conclusion, we could say that ethical approaches should not be reduced solely to a 'utilitaristic approach', i.e. a cost vs. benefit ratio, and

Copyright © 2003 John Wiley & Sons, Ltd. 

should take into account possible help coming from the scientific area. Most of all, we insist on the responsibility of the scientific community towards the public. Scientists should not decide on their own in matters, e.g. life issues, natural vs. engineered animals, that concern the majority of human beings. The paradox of the present matter is that the resurgence of questioning about the relationship between humans and animals emerges from the area of engineered life itself. A genomic approach used in order to improve philosophical reflection seems to be a good way to illustrate the link between genomics and bioethics.

### Acknowledgements

I would like to thank Jean-Paul Renard for his very constructive help during the preparation of the manuscript. I would also like to thank Magali Leroy and Martin Grana for their help during the re-reading.

### References

1. Ajmone-Marsan P, Valentini A, Cassandro M, *et al.* 1997. AFLP markers for DNA fingerprinting in cattle. *Anim Genet* **28**: 418–426.
2. Antoine S. 2000. *Protection de l'Animal*, Fasc. 1991 Editions du Juris-Classeur: Paris, France; 1–25.
3. Burgat F. 2002. La 'dignité de l'animal'; intrusion dans la métaphysique du propre de l'homme. *L'Homme* **161**: 197–204.
4. Chavatte-Palmer P, Heyman Y, Renard J-P. 2000. Cloning and associated physiopathology of gestation. *Gynecol Obstet Fertil* **28**(9): 633–642.
5. Colleau J-J, Heyman Y, Renard J-P. 1998. Les biotechnologies de la reproduction chez les bovins et leurs applications réelles ou potentielles en selection. *Product Anim* (INRA) **11**: 41–56.
6. Heyman Y, Chavatte-Palmer P, Le Bourhis D, *et al.* 2002. Frequency and occurrence of late-gestation losses from cattle cloned embryos. *Biol Reprod* **66**: 6–13.
7. Kang YK, Koo DB, Park JS, *et al.* 2001. Aberrant methylation of donor genome in cloned bovine embryos. *Nature Genet* **28**: 173–177.
8. Larrère C. 2000. Animal rearing as a contract? *J Agr Environ Ethic* **12**(1): 51–58.
9. Larrère R. 2000. Faut-il avoir peur du génie génétique? In *Nature. Les Cahiers Philosophiques de Strasbourg*, vol 10. Strasbourg; 11–48.
10. Lewis PD, Parry JM. 2002. An exploratory analysis of multiple mutation spectra. *Mutat Res* **518**(2): 163–180.
11. Ohgane J, Wakayama T, Kogo Y, *et al.* 2001. DNA methylation variation in cloned mice. *Genesis* **30**(2): 45–50.
12. Pellegrin P. 1982. *Les Classifications des Animaux chez Aristote. Statut de la Biologie et Unité de l'Aristotélisme*. Coll. D'études anciennes G. Budé. Les Belles Lettres, Paris.
13. Renard J-P, Qi Zhou, LeBourhis D. 2002. Nuclear transfer technologies: between successes and doubts. *Theriogenology* **57**: 203–222.
14. Rossant J, Spence A. 1998. Chimeras and mosaics in mouse mutant analysis. *Trends Genet* **14**(9): 358–363.
15. Sandoe P, Holtug N. 1998. Ethical aspects of biotechnology in farm animal production. *Acta Agric Sect A Anim Sci Suppl* **29**: 51–58.
16. Van Reenen CG, Meuwissen TH, Hopster H, *et al.* 2001. Transgenesis may affect farm animal welfare: a case for systematic risk assessment. *J Anim Sci* **79**(7): 1763–1779.
17. Vos P, Hogers R, Bleeker M, *et al.* 1995. AFLP: a new technique for DNA fingerprinting. *Nucleic Acids Res* **21**(23): 4407–4414.
18. Xiong LZ, Xu CG, Saghai Maroof MA, Zhang Q. 1999. Patterns of cytosine methylation in an elite rice hybrid and its parental lines, detected by a methylation-sensitive amplification polymorphism technique. *Mol Gen Genet* **261**(3): 439–446.
19. Yamamoto F, Yamamoto M, Soto JL, *et al.* 2001. *Not*1–*Mse*ll methylation-sensitive amplied fragment length polymorphism for DNA methylation analysis of human cancers. *Electrophoresis* **22**(10): 1946–1956.
20. Young LE, Sinclair KD, Wilmut I. 1998. Large offspring syndrome in cattle and sheep. *Rev Reprod* **3**: 155–163.

Copyright © 2003 John Wiley & Sons, Ltd. 
